# Understanding genetic variants in context

**DOI:** 10.7554/eLife.88231

**Published:** 2024-12-03

**Authors:** Nasa Sinnott-Armstrong, Stanley Fields, Frederick Roth, Lea M Starita, Cole Trapnell, Judit Villen, Douglas M Fowler, Christine Queitsch

**Affiliations:** 1 Herbold Computational Biology Program, Fred Hutchinson Cancer Center Seattle United States; 2 Department of Genome Sciences, University of Washington Seattle United States; 3 Brotman Baty Institute for Precision Medicine Seattle United States; 4 Department of Medicine, University of Washington Seattle United States; 5 Donnelly Centre and Departments of Molecular Genetics and Computer Science, University of Toronto Toronto Canada; 6 Lunenfeld-Tanenbaum Research Institute, Mt. Sinai Hospital Toronto Canada; 7 Department of Computational and Systems Biology, University of Pittsburgh School of Medicine Pittsburgh United States; 8 Department of Bioengineering, University of Washington Seattle United States; University of Pennsylvania United States; University of Sydney Australia

**Keywords:** gene–environment interactions, multiplexed assays of variant effect, epistasis

## Abstract

Over the last three decades, human genetics has gone from dissecting high-penetrance Mendelian diseases to discovering the vast and complex genetic etiology of common human diseases. In tackling this complexity, scientists have discovered the importance of numerous genetic processes – most notably functional regulatory elements – in the development and progression of these diseases. Simultaneously, scientists have increasingly used multiplex assays of variant effect to systematically phenotype the cellular consequences of millions of genetic variants. In this article, we argue that the context of genetic variants – at all scales, from other genetic variants and gene regulation to cell biology to organismal environment – are critical components of how we can employ genomics to interpret these variants, and ultimately treat these diseases. We describe approaches to extend existing experimental assays and computational approaches to examine and quantify the importance of this context, including through causal analytic approaches. Having a unified understanding of the molecular, physiological, and environmental processes governing the interpretation of genetic variants is sorely needed for the field, and this perspective argues for feasible approaches by which the combined interpretation of cellular, animal, and epidemiological data can yield that knowledge.

## Introduction

**Figure 1.**
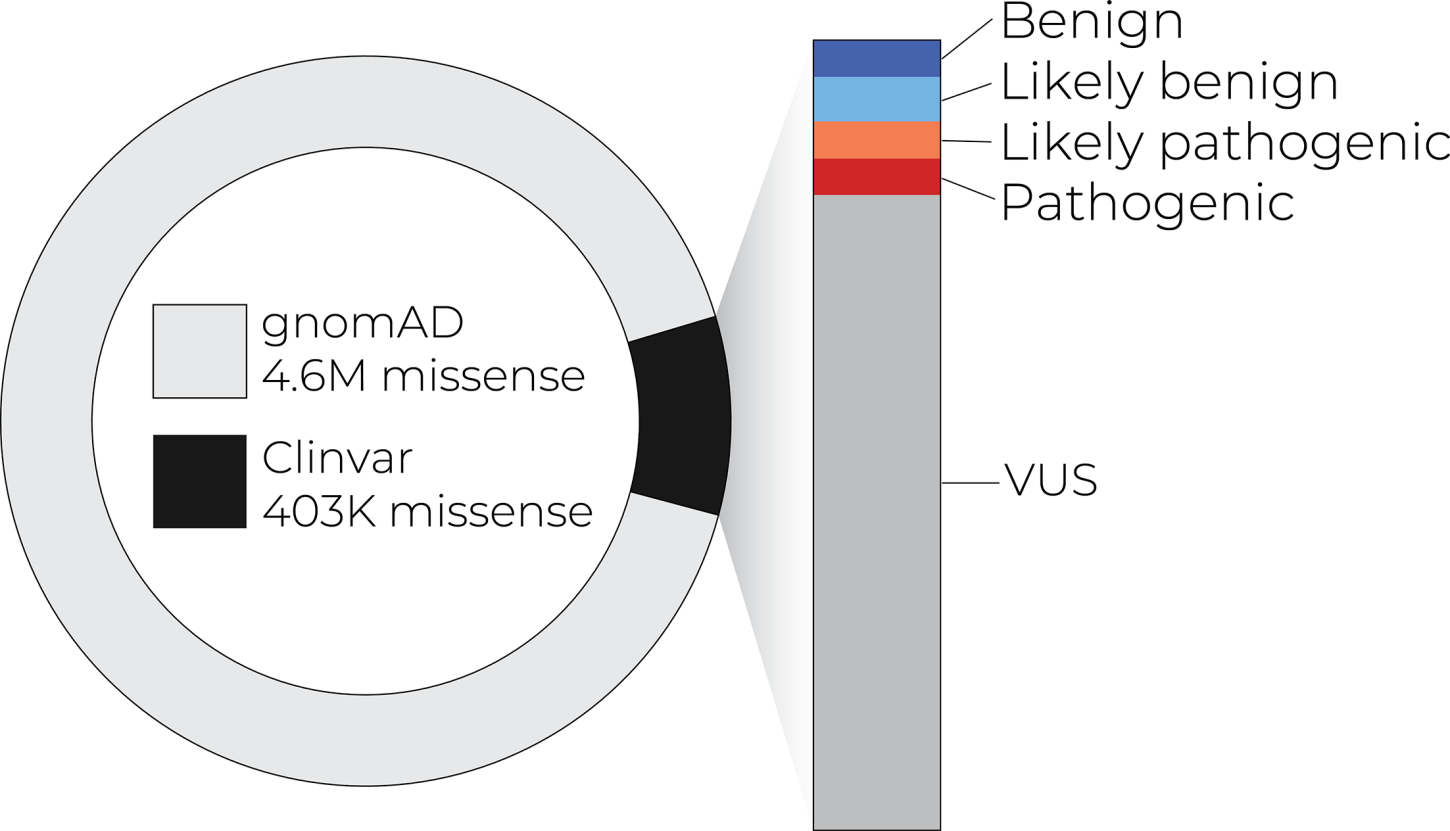
Only a small number of coding variants have annotations that can guide diagnosis and treatment. As exome and whole-genome sequencing becomes commonplace in the clinic, the number of variants of uncertain significance is likely to increase.

As a consequence of stunning technological advances – especially in DNA-sequencing – current databases hold hundreds of millions of human single-nucleotide variants, with nearly 5 million in the tiny portion of the genome that encodes protein sequence (Figure 1). Yet our knowledge of the functional effects of all this variation is vanishingly small: even for the changes that result in amino acid replacements, only about 2% have been clinically interpreted, and about 80% of those have been interpreted as ‘variants of uncertain significance’ (Fayer et al., 2021; Starita et al., 2017; Figure 1). Breaking through this interpretative bottleneck constitutes a central challenge for human genomics research.

Multiplex assays of variant effect (MAVEs) experimentally assess the effects of single-nucleotide variants at scale. Here, a single open-reading frame, exon, or regulatory region is saturated with all possible single-nucleotide changes, and a single property is measured via coupling this property to the number of DNA sequence reads of each variant before and after a functional selection (Esposito et al., 2019; Findlay, 2021; Kinney and McCandlish, 2019; Starita et al., 2017). We and others have shown that the resulting functional data can reveal whether and how each variant alters function, and that the functional data empower the interpretation of variants of uncertain significance (Fayer et al., 2021; Scott et al., 2022; Starita et al., 2017; Sun et al., 2016; Tabet et al., 2022; Weile et al., 2021; Wu et al., 2021; Yang et al., 2017). For example, the integration of multiplex functional data for cancer-related genes led to the reinterpretation of ~70% of variants of uncertain significance in *TP53*, ~50% in *BRCA1*, and ~15% in *PTEN* (Fayer et al., 2021). Spurred on by these and other results, the first generation of MAVEs is being deployed widely (Da et al., 2021; Esposito et al., 2019; Fowler et al., 2021; Rubin et al., 2021), and comprehensive variant effect maps for easy-to-measure cellular properties, such as growth, are within reach for many clinically relevant human genes.

**Figure 2.**
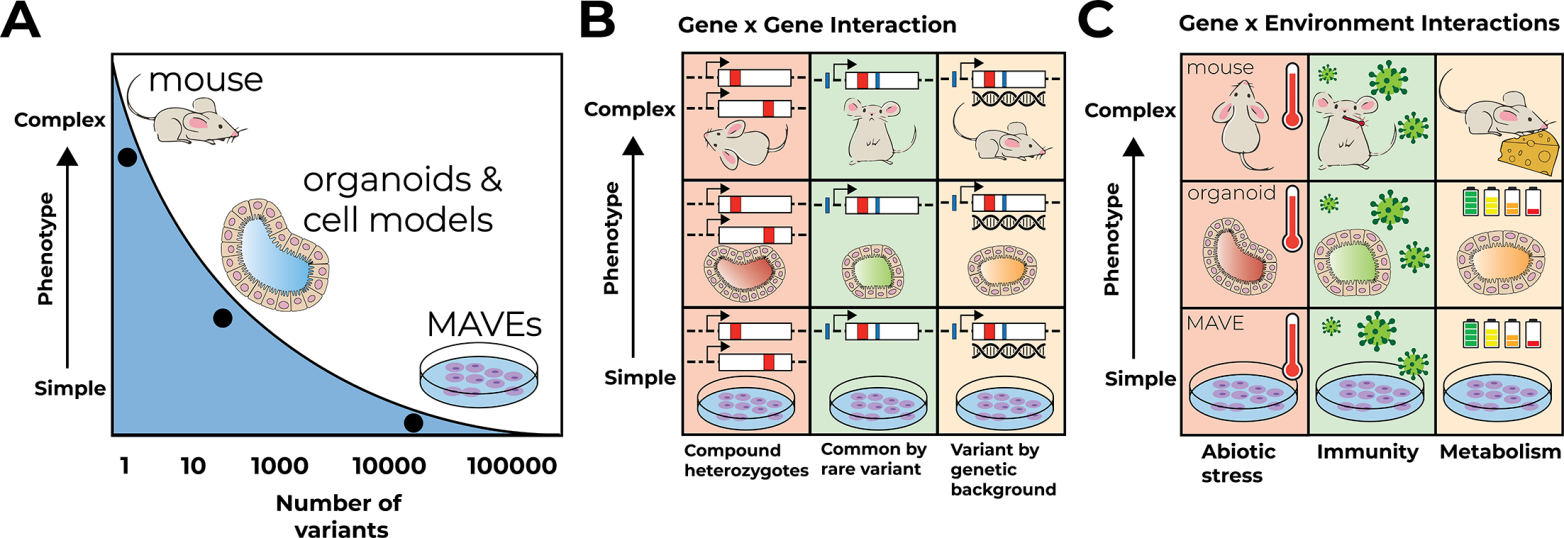
Multiplex assays of variant effect (MAVEs) in context. (**A**) MAVEs in cell lines can assay many variants for simple phenotypes like cell growth. Models like organoids and mice allow for measuring complex multicellular phenotypes like proportions of cell types but are currently limited to assaying only a few variants at a time. (**B**) Gene–gene interactions are examined in different models at different levels of phenotype complexity. Gene–gene interactions suggested for prioritization include compound heterozygotes, combinations of common and rare variants in a given locus in *cis* and *trans*, and experiments testing variants on different genetic backgrounds. (**C**) Gene–environment interactions are examined at different levels of phenotype complexity. Three broad categories are suggested to model the complexity of environmental context in the laboratory: abiotic stress, challenges to immunity, and metabolism.

Despite this success, we are far from being able to reliably interpret the organismal effects of all human genetic variation, much less to use genetic information to accurately predict individual phenotype and disease risk. For one, MAVEs have not been extended to structural variation, copy number variation in repetitive DNA, and other large and complex variants that are likely numerous and highly impactful (Manolio et al., 2009; Miller et al., 2021; Mitra et al., 2021; Press et al., 2014; Press et al., 2019). To make such complex variation accessible for phenotyping in high throughput, new experimental and computational approaches are needed. Yet even for single-nucleotide variants for which MAVEs exist or can be envisioned, major challenges for accurate variant effect interpretation remain. Existing MAVEs generally do not account for genetic, environmental, or tissue/developmental context (Figure 2). Assessing and perturbing this context is essential for fully characterizing the effect of a genetic variant (Claussnitzer and Susztak, 2021). This lack of context constrains the utility of MAVEs for understanding how variants interact with genetic background, affect non cell-autonomous phenotypes, and alter organismal phenotype in interplay with environmental factors. Here, we explore the importance of each of these contexts for understanding single-nucleotide variants and describe a next generation of MAVEs, applicable to both coding and regulatory variants, and computational approaches that, together, can generate contextually informative variant effect maps and predict individual disease risk.

## Variant effect mapping in genetic context

Onset, severity, and even incidence of disease can differ widely among carriers of a given disease-associated variant. This is particularly true for small and moderate effect variants that are associated with common diseases through genome-wide association studies (GWAS). The heritability explained by GWAS variants tends to be small, and these variants typically have little power to predict the disease risk of individuals (Eichler et al., 2010; Gibson, 2012; Khera et al., 2018; Manolio et al., 2009). This ‘missing heritability’ of common diseases and the differences in variant expressivity among patients with highly penetrant, rare Mendelian disorders are commonly attributed to uncharacterized non-additive genetic interactions, among other factors (Eichler et al., 2010; Gibson, 2012; Manolio et al., 2009).

Non-additive genetic interaction, or epistasis, describes the phenomenon that the combined effect of alleles at two or more loci deviates from the sum of their individual effects (Fisher, 1919; Mackay, 2001). The importance of non-additive genetic interactions, in particular for complex traits, has been the subject of a long-standing debate (Fisher, 1930; Hivert et al., 2021; Wade and Goodnight, 1998; Wright, 1931), and the importance of epistasis across the allele frequency spectrum is not well understood. Model organism research has identified many examples of non-additive genetic interactions affecting a wide variety of morphological and quantitative traits in fungi, animals, and plants (Costanzo et al., 2019; Forsberg et al., 2017; Mackay, 2014; Press et al., 2014). These genetic interactions often reflect functional relationships such as those among genes coding for subunits of a multimeric protein complex or proteins functioning in a common pathway (Costanzo et al., 2019; Miller and Piccolo, 2020; Narasimhan et al., 2017; Ni et al., 2017; Pamplona-Cunha et al., 2020). Quantitative genetics theory and empirical data for human and crop traits show that additive genetic models can explain over half of the total genetic contributions to complex traits (Darnell et al., 2022; Dudley, 2007; Hivert et al., 2021; Sheppard et al., 2021; Visscher et al., 2008). However, accounting for non-additive genetic effects, and particularly epistatic interactions, can lead to more accurate phenotype predictions (Carlborg et al., 2006; Forsberg et al., 2017; Lachowiec et al., 2015; Matsui et al., 2022). Methods that examine genetic ancestry and portability can also improve prediction and estimation in the presence of these effects (Brown, 2016; Park et al., 2018; Patel et al., 2022).

Because a large majority of disease-associated GWAS variants reside within or in linkage with accessible chromatin regions, complex diseases are assumed to arise through the additive action of many regulatory variants (Maurano et al., 2012; Meuleman et al., 2020). The enrichment of GWAS variants in or near regulatory regions, their large numbers, and their small contribution to heritability and disease risk were conceptualized in the ‘Omnigenic’ model (Boyle et al., 2017; Liu et al., 2019). This theoretical framework posits that ‘core genes’, which are functionally related to a phenotype of interest, carry common, small-effect, significantly trait-associated variants that together contribute little to heritability while the bulk of heritability is contributed by a huge number of common ‘peripheral’ variants with individually tiny effects that ultimately affect core gene expression. Peripheral variants are uniformly distributed over the genome, with each chromosome contributing to trait heritability according to its size, reminiscent of Fisher’s Infinitesimal model (Barton et al., 2017; Walsh and Lynch, 2018). The Omnigenic model is not universally accepted (Connally et al., 2022; Sohail et al., 2019), but it offers an explanation for the missing heritability conundrum and is supported by recent empirical studies (Sinnott-Armstrong et al., 2021; Smith et al., 2022). If most heritability is not located at genes or regulatory regions directly altering the phenotype of interest, how can we use experimental variant effect mapping in context to understand disease risk? In any case, there are several ways that experimental variant effect mapping in context can be applied to understand disease risk.

An obvious approach is systematically studying compound heterozygotes (Figure 2B). There are many recessive variants of unknown molecular or cellular functional consequence in disease-associated genes, even amongst the thousands of genes that play a role in highly penetrant Mendelian disorders. Examples abound of cases in which individuals carry different alleles of a gene that together – as a compound heterozygote – alter gene activity enough to cause disease. These alleles may act additively or non-additively, enabling valuable mechanistic insights on disease origins, a first crucial step toward future therapy. Another promising approach is assaying combinations of common, disease-associated GWAS variants with all possible other variants in the same gene and regulatory region (Figure 2B), a likely scenario by which the penetrance and expressivity of a common variant may be altered. To address both of these scenarios, variants of a given gene or regulatory region will need to be assessed in pairwise fashion. Executing such experiments in human cells across all possible variant pairs will require some innovation in variant barcoding, genome engineering, and sequencing strategies to unambiguously link both variants with a cell’s phenotype. However, the systematic evaluation of compound heterozygotes and of combinations of GWAS variants with rare variants affecting the same locus will yield actionable information for genetic counselors, physicians and patients.

Experimentally addressing the consequences of more complex genetic interactions (Figure 2B) will require sophisticated technological innovations and novel data analysis methods. For example, variant libraries could be introduced into many different cell lines, including induced pluripotent stem cells (iPSCs) derived from diverse individuals, thereby testing the consequences and interactions of many different genetic variants, including structural variants. Identifying causal additive or non-additive genetic interactions in this scenario will be a formidable challenge. Alternatively, variant libraries could be introduced into cells carrying programmed genetic perturbations such as gene deletion, knockdown, or overexpression. Most ambitious, variant libraries for a disease-associated core gene could be introduced into cell lines carrying common variants in other core genes for the same trait, testing some assumptions of the Omnigenic model empirically. If it were possible to conduct variant effect mapping in whole animals, genetic crosses and high-throughput phenotyping could be used to interrogate the phenotypes of many different combinations of variant libraries with each other, other variant libraries, or possible modifier loci.

Most importantly, these experimental efforts must go hand in hand with innovative theoretical studies to enable predictions of variant effects in genetic context. For example, combining existing variant effect maps of disease-associated genes with genome-wide polygenic risk scores is one approach to investigate the Omnigenic model. Such a modeling approach could leverage the growing resources of biobanks and human phenotype data to identify modifier loci and refine variant effect maps (Schiabor Barrett et al., 2022; Tabet et al., 2022). Similarly, taking advantage of the multitude of high-quality GWAS, one could systematically explore loci that are significantly associated with different disorders; these loci represent candidates for genetic modifiers that alter the penetrance and expressivity of other variants (Lehner et al., 2006; Queitsch et al., 2012). One such locus is known: the major histocompatibility complex (MHC), which encodes cell surface proteins that are essential for the adaptive immune system by virtue of their ability to ‘display’ a cell’s repertoire of contained peptides to the immune system. The fact that different MHC alleles display different sets of variant peptides offers avenues for MHC variation to alter the penetrance and expressivity of many other variants. Stratifying existing GWAS by outliers, for example*,* focusing on individuals with the most and least severe disease phenotypes, might also identify genetic modifiers of variant penetrance and expressivity, where they exist (Gibson, 2009; Lehner et al., 2006; Queitsch et al., 2012). Although there is limited evidence for dominance in human genotype–phenotype mapping and epistasis remains difficult to quantify genome-wide thus far, we anticipate that understanding epistatic effects at individual genes will yield valuable insight into the extent of these epistatic interactions and their phenotypic consequences.

## Variant effect mapping in cell, tissue, and developmental contexts

Although variants in some genes affect all cell types similarly across development, variants more often exert their effect in specific tissues, and at specific stages of development. For example, pathogenic germline variants in *BRCA1* and *BRCA2* genes, which play a fundamental role in DNA repair, confer a greatly increased risk of some, but not all, types of cancer (Welcsh and King, 2001). Variants affecting metabolic or neurodevelopmental disorders like atypical pantothenate kinase-associated neurodegeneration and schizophrenia can show effects in specific tissues and/or developmental stages (Immonen et al., 2017; Kurian and Hayflick, 2013). Moreover, different variants in the same gene can affect different tissues and cause different diseases. For example, pathogenic germline variants in *LMNA* most often cause cardiomyopathy, but can also cause muscular dystrophy, lipodystrophy, or the premature aging syndrome progeria (Novelli and D’Apice, 2003). This complexity only expands with regulatory variants at a given locus, where enhancer elements are able to activate multiple genes (Hu and Tee, 2017), causing pleiotropic effects on tissue specificity. While recent approaches have dramatically improved the linkage of regulatory variants to target genes (Andersson et al., 2014; Dey et al., 2022; Gschwind et al., 2023; Nasser et al., 2021; Kundaje et al., 2015), this remains an area of substantial ongoing work. Even so, numerous existing studies have begun to tackle the application of MAVE technologies to regulatory regions (Gasperini et al., 2016; Gordon et al., 2020; Kircher et al., 2019; Klein et al., 2018; Morova et al., 2023; Vockley et al., 2015). These studies rely on the same assumptions as coding variant MAVEs, namely that there is a direct link to the phenotype being selected and that variants have variable effects on that phenotype in the cell type being screened.

Tissue- and developmental stage-specific effects cannot be addressed in human cell lines that lack the expression programs, proteomes, and cellular structures found in fully differentiated cells. However, most MAVEs have been executed in human cell lines and have focused on easily screenable phenotypes like cell growth, protein abundance, or protein–protein interactions. Recent efforts to develop MAVEs based on rich phenotypes like cell morphology or transcriptional programs have demonstrated the potential of investigating these more complex phenotypes (Hasle et al., 2020; Martin-Rufino et al., 2023; Ursu et al., 2022; Xu et al., 2023). Disentangling the relationships between phenotypes is challenging, primarily as a result of pleiotropy (Solovieff et al., 2013). However, MAVEs test the entire set of possible alleles in a gene or regulatory region, allowing us to determine the full spectrum of allele-specific effects on phenotypes that can be modeled in a scalable assay.

Moreover, researchers have begun to explore iPSC-derived differentiated cells to model variant effects (Bajpai et al., 2021). However, owing to the challenges in engineering iPSC genomes, MAVEs in these cells are in their infancy (Lv et al., 2018). A potentially more powerful approach would be to isolate specific primary cell populations from individual patients (Claussnitzer et al., 2015; Shifrut et al., 2018), introduce variant libraries, and ascertain variant effects on cellular phenotypes. Complementing these efforts with other approaches like base or prime editing (Erwood et al., 2022; Hanna et al., 2021) could lead to the characterization of large variant libraries in the correct cellular context. Successful proof-of-principle studies demonstrate the potential of this approach (Martin-Rufino et al., 2023). However, these approaches are predicated on knowing the correct cell type(s) for the phenotype of interest. This information is often not available, but deriving robust cell type–disease associations is the subject of significant ongoing work (Tabet et al., 2022; Jones et al., 2022). Recent computational methods have also opened the possibility of accurate estimation of cell types of action in silico (Jagadeesh et al., 2022; Yu et al., 2022).

However, even differentiated cells do not recapitulate cell–cell interactions found in human tissues, much less the complex ballet of interactions required for normal development (Dorrity et al., 2022; Saunders et al., 2022). To model cell–cell communication will require multicellular models. Organoids, especially those derived from patient cells, offer a possible solution as they can model various types of tissues (Hendriks et al., 2021; Meng et al., 2022). The main challenges here are developing genome engineering approaches that can yield a large set of clonal organoids while accounting for the sometimes large phenotypic variation among organoids of the same type generated with the same protocol; such efforts are in progress (Sockell et al., 2022).

Model organisms are another option. Multicellular model organisms from worms to mice have proven extraordinarily useful in probing the tissue and developmental effects of individual genetic variants. However, to map variant effects at large scale in a model organism, it must produce large numbers of offspring to allow for adequate coverage of variants and high-throughput phenotypic screens of developmental or behavioral traits; The ideal organism would offer strong conservation of genes and pathways affected in human disease. Model organism approaches are particularly powerful when paired with single-cell transcriptomic readouts, as was used to investigate the consequences of 23 genetic perturbations affecting the development of cell lineages in zebrafish (*Danio rerio*) (Saunders et al., 2022). Leveraging single-cell transcriptomics, large numbers of replicates, and sophisticated statistical analysis, this study ascertained the consequences of a particular perturbation on the variance in cell type abundance organism-wide and detected the perturbation-dependent effects on cell type composition relative to wild-type embryos. Theoretically, single-cell genomics could be applied to a large number of small animals, each expressing a single variant or variant combinations, to similarly determine the consequences of genetic variants on cell type composition, gene expression, chromatin accessibility, protein abundance, and other single-cell phenotypes.

However, to enable MAVEs in whole animals, new technology is needed. The biggest challenge is that variant libraries must be introduced into animals such that thousands or more variants are represented across many thousands of animals. Germline editing is preferred to generate distinct, clonal recombinant animals, avoiding mosaicism and easing phenotype interpretation. Considering currently available genetically tractable models and their offspring numbers, the few in which such technology development seems worthwhile include *Caenorhabditis elegans*, *Drosophila melanogaster,* and *Danio rerio*. These animal models lend themselves readily to ascertaining the phenotypes of compound heterozygotes and variant combinations in different genes through crosses. However, it remains a formidable, unsolved challenge to successfully introduce large variant libraries into an animal germline such that each genome contains just one variant, each variant is present in many animals for replication of variant effects, and each variant is expressed at the same level.

## Variant effect mapping in environmental context

Environmental context (gene–environment interactions) plays a large role in variant penetrance and expressivity, particularly for variants associated with common diseases such as diabetes, asthma, depression, and cardiovascular disease. Their incidence has risen sharply in recent decades in the United States and elsewhere (Bingley and Gale, 1989; Cockram, 2000; Ebrahim et al., 2010; Gibson, 2009; Harsanyi et al., 2022; Rewers et al., 2018; Sørensen, 2000). Because human genetic makeup has not fundamentally changed in the last 50 years, changing environmental context has either altered the genetic contributions of a subset of polymorphisms or shifted the liability threshold for these disorders. Fundamental recent changes to environmental context include altered pathogen exposure and microbiomes through increased hygiene, refrigeration, and antibiotics; radical dietary shifts toward industrially produced food; and environmental stress through artificial light, house dust, and harmful chemicals. Although we posit that environmental context is more crucial than genetic or developmental context for understanding variant penetrance and expressivity, environment is also the hardest context to fully define and measure in the laboratory.

The challenges of exploring environmental context in humans are well illustrated by the TEDDY study (The Environmental Determinants of Diabetes in the Young) (Rewers et al., 2018). The study followed ~9000 children who carry high-risk alleles for type I diabetes for 15 years, collecting clinical metadata (e.g., diet, household exposures, medications, pre- and perinatal exposures, psychosocial stressors, among many others), and the results of many ‘omics’ analyses (e.g., whole-genome sequencing, metabolomics, microbiome, lipidomics, transcriptomics, proteomics). One challenge was participant dropout (26%) due to the high participation effort (Johnson et al., 2011; Johnson et al., 2014). Another was the relatively small number of children who ultimately developed type 1 diabetes (~300). These logistical challenges limited the study’s power to detect gene–environment interactions. The published results suggest little effect of many environmental factors such as breastfeeding (Hummel et al., 2021), early antibiotic treatment (Kemppainen et al., 2017), vaccinations (Elding Larsson et al., 2018), and maternal exposures (Johnson et al., 2021b; Johnson et al., 2021a; Silvis et al., 2019), while a few studies report evidence for possible risk factors (infection) (Lönnrot et al., 2017; Vehik et al., 2019) or interventions (vitamin D, probiotics) (Norris et al., 2018; Uusitalo et al., 2016).

**Figure 3.**
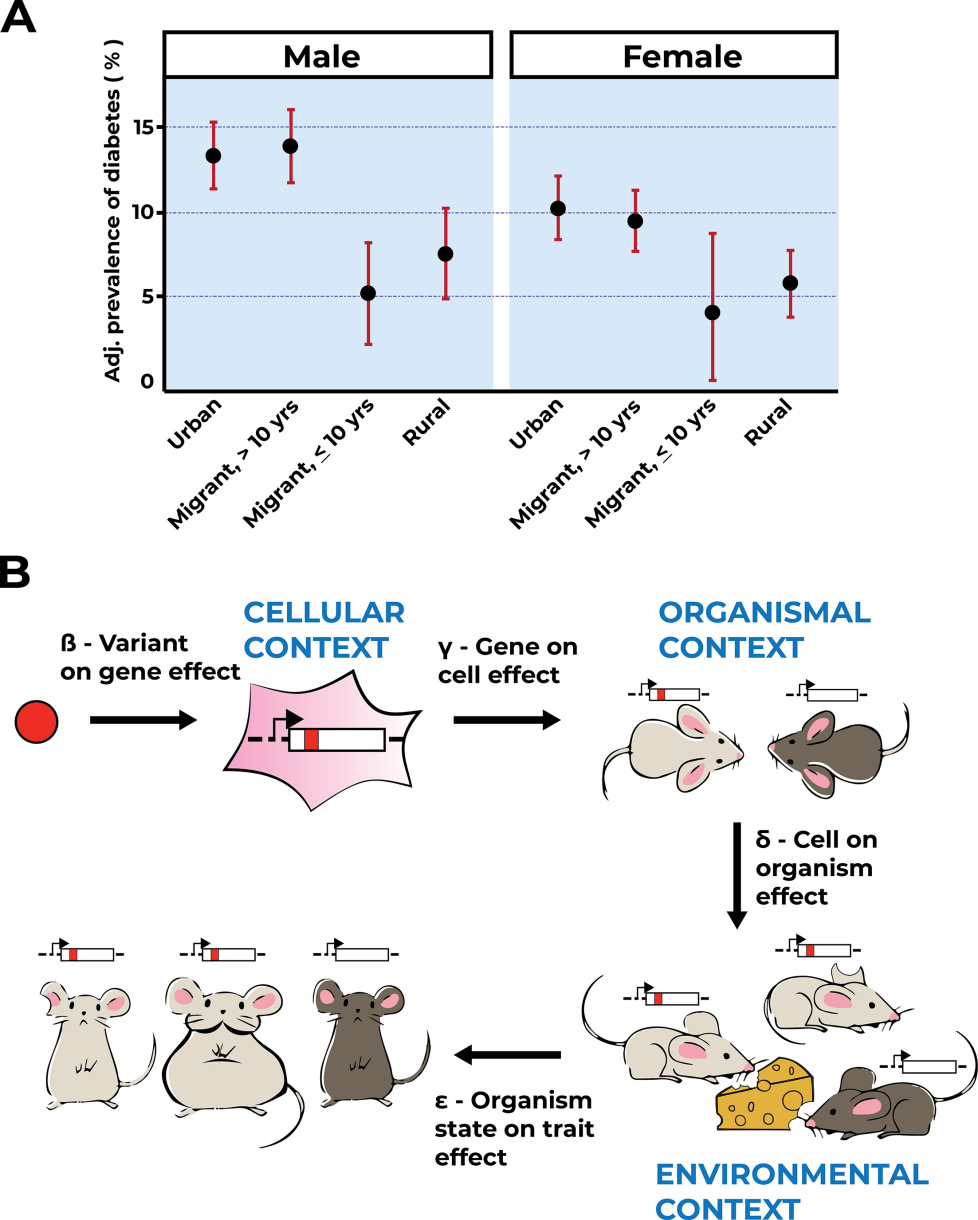
Environmental context is key to trait interpretation. (**A**) Adapted from Figure 1 of Ebrahim et al., 2010, age-, factory-, and occupation-adjusted percent prevalence (95% CI) of diabetes by type of migrant and sex, Indian migration study 2005–2007. Diabetes is prevalent in urban residents and residents who migrated to urban areas and resided there for more than 10 years. (**B**) Gene–environment interactions will affect an organismal trait at the level of genes, cells, tissues, and whole organisms. Extending the Mostafavi et al., 2022 model to incorporate environmental context captures more relevant biology, and hence facilitates variant effect interpretation. As shown, a variant (red allele) affects a gene’s function within a particular cellular context. Cells affected by the red allele function exist within the context of the organism, here a light-gray mouse as compared to a dark-gray mouse that does not carry the red allele. Mice with or without the red allele are exposed to environmental factors, symbolized by the cheese as a dietary factor challenging metabolism. Note that one of the mice carrying the red variant is not exposed to this environmental challenge (light gray, clipped ear). In the example shown, environmental context determines the trait value (obesity, big light-gray mouse) for mice carrying the red variant.

While it may be difficult to pinpoint relevant gene–environment interactions across large human populations, these studies nevertheless hold much promise, in particular when considered together with the results of experimental variant effect maps. For example, a dense variant effect map for a particular gene or regulatory region produced in a single condition could be supplemented with human phenotype data for carriers of particular variants. These individuals will have experienced a large range of environmental conditions, beginning in utero and continuing after birth. To estimate their gene–environment interactions, one would have to account for (potentially non-additive) genetic background, which can be accomplished through sibling study designs, kinship analyses, or use of polygenic risk scores (Mostafavi et al., 2020). The ever increasing size (i.e*.,* sample number) and sophistication (i.e., inclusion of multiple traits, ancestries, and environmental exposures) of today’s GWAS make such an approach imminently feasible, though the approach might still be insufficient for traits with large epistatic components in their genetic architecture. Large sibling and twin cohorts collected over many decades may also aid in computational efforts to decipher the effect size of gene–environment interactions and the variants most affected. As siblings and twins share (to varying degrees) both their genome and their environment, increasing concordance of disease incidence and severity would be expected for relatives carrying genetic variants associated with moderate or low disease risk in GWAS and implicated as pathogenic or likely pathogenic in functional assays. If gene–environment interactions have changed, family studies will allow extrapolation of gene–environment effect size, if not necessarily identification of responsible environmental factors. The same approach can be applied in reverse using migration studies and other approaches for causal effect estimation from the social sciences to further isolate gene–environment effects (Lea et al., 2023; Figure 3). This can include direct approaches for modeling gene–environment interactions (Kerin and Marchini, 2020; Marderstein et al., 2021; Moore et al., 2019), as well as integration of causal effect size estimation using quasi-experimental approaches (Barcellos et al., 2018; Barcellos et al., 2021; Davies et al., 2018; Plotnikov et al., 2020; Abdellaoui et al., 2019; Ebrahim et al., 2010; von Hinke and Sørensen, 2023).

Efforts to determine gene–environment interactions with MAVEs are nascent. An example is a recent collection of variant effect maps for *MTHFR*, encoding a key enzyme in folate metabolism. MTHFR deficiency can be severe, with diverse early-onset consequences of a massive accumulation of homocysteine in the blood, or relatively mild, with later-emerging thromboembolism. Milder MTHFR deficiency can be remediated for some patients by increasing folate levels. Still milder effects result from homozygosity of the common Ala222Val variant (30% global minor allele frequency), for which there is a risk of neural tube defects that is entirely remediated by sufficient dietary folate. Thus, the pathogenicity of variants is dependent on environmental context. This dependency of variant effect on the environmental context of folate levels (and on the genetic context of an A222V variant) was captured for nearly every *MTHFR* missense variant (Weile et al., 2021). Other examples include efforts to account for proteotoxic stress, for example, by evaluating transcription factor variants at elevated temperature or in response to chaperone inhibitors (Dorrity et al., 2018; Morton et al., 2020). None of these examples yet captures the full complexity of environmental context given that only a few environmental variables were each altered one at a time.

Addressing environmental context has to grapple with the overwhelming number of possible environmental factors that could alter a variant’s impact. For some genes, relevant environmental factors are known and these factors can be directly incorporated into multiplexed assays, as for *MTHFR*. Even for *MTHFR*, however, there is evidence that levels of riboflavin, as well as folate, can also remediate the effects of the common Ala222Val variant (Jarrett et al., 2022; McNulty et al., 2006), and the interactions across the broader set of *MTHFR* variants alone or in combination with folate and Ala222Val remain to be explored. More broadly, the profound changes in environmental context in the last 50–100 years suggest a focus on three broad categories: environmental factors altering metabolism, causing abiotic stress, and challenging the immune system. These broad categories can be modeled (with increasing degrees of difficulty) in the laboratory and studied both individually and in combination. Combinatorial studies are essential as responses to individual perturbations are often not predictive of responses to combinations, at least in model organisms (Ammeux et al., 2016). We envision environmental perturbations in cell lines, organoids, and even model animals carrying variant libraries (Figure 2C).

In addition to variant effect mapping across a range of external conditions, the internal conditions of the cells and organisms carrying variants can be drastically changed. The relative importance of external versus internal cellular environments is much debated (Billman, 2020). Here, an approach to induce mistranslation across the entire proteome for a given codon (Berg et al., 2019; Cozma et al., 2022) can manipulate internal cellular environments and allow their effects on individual variants to be measured. Similarly, one may consider manipulating splicing, protein folding, protein turnover, or mitochondrial function as general measures to affect cellular environment as it occurs in human aging (Johnson et al., 1999; Leutert et al., 2023). Analogously, pharmacological perturbation of key cellular processes could be applied to alter cell–cell interactions and niche formation to understand intra-organismal (i.e., organism-autonomous) effects of genetic variants.

Quantifying gene–environment effects on an organismal trait must consider prior knowledge of variant effects on gene function, the consequences of perturbed gene function on cellular function within the context of the whole organism, and an organism’s environmental exposures (Figure 3B). These multiple unknown factors are difficult to disentangle without acquiring knowledge of each component, but data from MAVEs enable us to do so systematically. Similar to pleiotropy (Solovieff et al., 2013), gene–environment interactions will affect an organismal trait at the level of genes, cells, tissues, and whole organisms (Figure 3B). Under the simplifying assumption that most genetic effects are due to variants acting on genes or their products, we can separate variant effects on a trait into two components, the contribution of that variant to the target gene and the effect of that gene on the trait of interest (Mostafavi et al., 2022). Incorporating environmental context into this model makes it substantially more complicated, but more reflective of biological reality, and hence more predictive of variant effects and disease risks (Figure 3B). The scale and highly controlled nature of MAVE-derived variant effects will make it possible to disentangle the contributions of environmental interactions from the two components as defined above.

## Multiplex assays of variant effect in context to decipher mechanisms, improve disease risk predictions, and facilitate prevention and treatment

In the clinic, the individual matters. Each of us has our private set of common and rare variants and our unique environmental exposures that together determine our disease risk and prognosis. We argue that MAVEs in genetic, developmental, and environmental contexts will allow a systematic characterization of the relative importance of each context for variant effect as well as the characterization of variants particularly impacted by a given context or context combination. Moreover, as MAVEs that can measure phenotypes more complex than cell survival or cell fitness are deployed widely, we will begin to gather mechanistic insight on variant function at large scale and in response to multiple environmental perturbations. Current approaches to decipher biological mechanisms of specific variants through variant-to-function analyses remain laborious and highly specialized (Claussnitzer and Susztak, 2021; Tabet et al., 2022). As we must pursue mechanistic insights into variant effects to separate confounding from causal association, conducting MAVEs in context offers the promise of scale and systematic analysis. The more contextual multiplex variant functional data becomes available, the more modeling approaches that rely on vast datasets such as machine learning and artificial intelligence can be used to infer variant-specific and context-dependent biological mechanisms, thereby informing individualized predictions of disease risk for rare and common diseases and facilitating their prevention and treatment.

A key question is which genes and regulatory regions are most likely to yield the most useful information for precision medicine, and therefore should be the focus of MAVEs in context. A simple answer is to focus on the genes that contain many variants of uncertain significance and that are actionable with existing prevention and treatment options. This approach, however, will exclude many of the regulatory and coding GWAS variants associated with common disease for which context, in particular environmental context, will likely matter the most. If we are to understand complex common diseases, experimentalists and theoreticians will need to come together to innovate and develop strategies to study human variants at genome scale and in multiple contexts.

## Appendix 1

### Glossary of the terms used herein

#### Variant of uncertain significance

A genetic variant that has been identified but whose significance to disease etiology and health of an individual is not known. These variants cannot guide diagnosis or treatment.

#### Heritability

An attribute of a quantitative trait in a population that expresses how much of the observed total phenotypic variation is due to genetic variation. Narrow-sense heritability describes the fraction of phenotypic variation due to the additive effects of genes. Broad-sense heritability captures the fraction of phenotypic variation due to genetic values that may include non-additive effects.

#### Dominance

In human genetics, this usually refers to alleles that manifest their phenotype in the heterozygous state.

#### Epistasis

In human genetics, this usually refers to non-additive genetic interaction and describes the phenomenon that the combined effect of alleles at two or more loci deviates from the sum of their individual effects.

#### Pleiotropy

Describes the phenomenon in which a single gene is responsible for a number of distinct and seemingly unrelated phenotypic effects.

#### Liability

A term used to collectively describe all the genetic and environmental factors that contribute to the development of a complex disease or trait. A large number of factors are summed to yield a 'liability' curve with a threshold marking a binary outcome (e.g., unaffected or affected by a disease). Phenotypic outcome is determined by whether an individual’s liability is smaller or greater than the threshold.

#### Variant penetrance

Refers to the proportion of individuals carrying a particular genetic variant with the expected phenotype(s) commonly associated with that variant.

#### Variant expressivity

Refers to the range and severity of phenotypes associated with a given genetic variant in different genetic and environmental contexts.

#### Rare genetic variants

Defined as variants with a minor allele frequency of less than 0.01.

#### Common genetic variants

Defined as variants with a minor allele frequency of 0.01 or greater.

#### Mendelian disease

Disease that follows Mendelian patterns of inheritance due to alterations in one gene or regulatory region inherited from one (dominant disorder) or both parents (recessive disorder). Mendelian diseases are often highly penetrant; although many such disorders are individually rare, together, they affect an estimated 5% of the world’s population (Rahit and Tarailo-Graovac, 2020).

#### Common complex disease

Common complex diseases lack simple Mendelian patterns of inheritance. These disorders are thought to be a consequence of genetic variation in many loci and environmental factors, whose interplay remains little understood and difficult to study.
